# 

**Published:** 1870-12

**Authors:** 


### Notices of Literary Exchanges.

The *Nation* for the coming year promises to be of greater interest and worth than ever. It will contain contributions from a great variety of sources.——The *Atlantic Monthly* presents a prospectus for the next year of as an attractive a character as usual. Several new and attractive features are to be added this year, and the departments of Science and Art are to be largely represented in it, by distinguished and learned men.——*Every Saturday* has come to be one of the first of the illustrated journals of the country; and while its reading matter is fresh, vigorous and entertaining, the character of many of the engravings it presents is that of surpassing excellence. The regular receipt of the issues of this paper cannot but be much desired.——*Littell's Living Age* continues to furnish weekly selections from the European journals of such articles as are of sterling worth and popular interest. It is energetically conducted, and many very excellent articles which it presented during the year are fresh in our recollection.——The *American Exchange and Review*, published by Fowler & Moon, of Philadelphia, is a miscellany of useful knowledge. It pays especial attention to art, science and trade. The original articles it contains are on subjects of the first interest to intelligent business men; and its several departments are separately edited by men skilled in the particular department they represent.——The *Scientific American* continues to be a great source of information and intelligence as to the progress of science and invention in this country and in other countries. Its editors are ever ready to do all in their power for the progress of invention and the promulgation of the facts of scientific discovery; and they thus recommend themselves and their publication to the public generally.——The *Little Corporal*, published in Chicago by Sewell & Miller, is one of the brightest and most entertaining of the magazines for "young folks" that comes to our table. Its subscription price is only one dollar a year, and the whole character of the publication is remarkably excellent.——*Peters' Musical Monthly* is the title of a journal of music issued every month by J. L. Peters, 599 Broadway, New York. Its subscription price is three dollars per year: the music it contains (36 pages per number) could not be otherwise obtained for many times this amount; and the numbers we have examined contain very desirable and excellent pieces.

### Books and Pamphlets Received.

Galvano-Therapeutics—Physiological and Therapeutical action of the Galvanic current upon the Acoustic, Optic, Sympathetic and Pneumogastric Nerves. By William B. Neftel, M. D. New York: D. Appleton, &c. For sale by Breed & Lent.

The Physics and Physiology of Spiritualism. By Wm. A. Hammond, M. D. New York: D. Appleton & Co.

Annual Report of the Surgeon General of the United States Army for 1870.

Report on the Progress of Ophthalmology, made to the American Ophthalmological Society, July, 1870. By B. Joy Jeffries, A. M., M. D., Boston, Mass.

Physical Degeneracy. By Nathen Allen, M. D., Lowell, Mass.

The Physiological Laws of Human Increase. By Nathan Allen, M. D., Lowell, Mass.

Partial Paralysis from Reflex Irritation, caused by Congenital Phimosis and Adherent Prepuce. By Lewis Sayre, M. D. New York City.

Histological Contribution. By H. G. Piffard, M. D., Surgeon to the New York Dispensary for Diseases of the Skin. Reprinted from the American Journal of Syphilography.

Introductory Lecture at Bellevue Hospital Medical College, Oct. 12th, 1870. By Frank H. Hamilton, M. D. New York City.

The Relations of the Medical Profession on Modern Education. By Edward S. Dunster, M. D. From the New York Medical Journal, Dec., 1870.

Illustrated Annual of Phrenology and Physiognomy for 1871. By S. R. Wells, Editor of Phrenological Journal.

Old Franklin Almanac for 1871. A. Winch, Philadelphia.

Vick's Illustrated Catalogue and Floral Guide for 1871; James Vick, Rochester, N. Y.

LIST OF EXCHANGES:—

American Journal of Medical Science; Archives of Ophthalmology and Otology; American Journal of Syphilography and Dermatology; American Journal of Obstetrics; American Practitioner; Baltimore Medical Journal; Baltimore Medical Bulletin; Boston Medical and Surgical Journal; California Medlcal Gazette; Canada Lancet; Cincinnati Lancet and Observer; Cincinnati Medical Repository; Chicago Medical Examiner; Chicago Medical Journal; Chicago Medical Times; Detroit Review of Medicine and Pharmacy; Dominion Medical Journal; Gynæcological Journal; Half Yearly Compend of Medical Science; Half Yearly Abstract of Medical Science; Indiana Journal of Medicine and Surgery; Journal of Insanity; Journal of Cutaneous Medicine and Surgery, Belfast; Leavenworth Medical Herald; London Lancet; Medical Record; Medical News and Library; Medical Times; Michigan University Medical and Surgical Journal; Nashville Medical and Surgical Journal; New Orleans Journal of Medicine and Surgery; New York Medical Journal; New York Medical Gazette; Northwestern Medical and Surgical Journal; Oregon Medical and Surgical Reporter; Pacific Medical and Surgical Journal; Philadelphia Medical and Surgical Reporter; Photographic Review of Medicine and Surgery; Psychological Journal; Richmond and Louisville Medical Journal; St. Louis Medical Archives; St. Louis Medical and Surgical Journal; Transactions of the College of Physicians and Surgeons of Philadelphia; American Eclectic Review; American Journal of Homoeopathic Materia Medica, Canada Health Journal; Cincinnati Eclectic Medical Journal; Medical Independent; Medical Investigator; New England Medical Gazette; Occidental; Ohio Medical and Surgical Reporter; Philadelphia Eclectic Medical Journal; U. S. Medical and Surgical Journal; American Journal of Dental Science; Canada Journal of Dental Science; Dental Register; Dental Cosmos; Dental Times; Dental Office and Laboratory; Missouri Dental Journal; St. Louis Dental Journal; American Chemist; American Journal of Pharmacy; Archives of Science; Boston Journal of Chemistry; Canadian Pharmaceutical Journal; Druggists' Circular; Journal of Materia Medica; London Chemist and Druggist; The Pharmacist; Atlantic Monthly: American Agriculturist; American Exchange and Review; American Messenger and Botschaft; American Educational Monthly: American Journal of Microscopy; Atlantic Beacon; Avon Journal; Child's Paper, Cosmopolitan; Every Saturday; Good Health; Hall's Journal of Health; Herald of Health; Le Citoyen Americain; Littel's Living Age; Little Corporal; New York Observer; Pastor and People; People's Literary Magazine; Steiger's Literarischer Monatsbericht; The Nation.

